# Lipidomics analysis unravels the effect of nitrogen fertilization on lipid metabolism in tea plant (*Camellia sinensis* L.)

**DOI:** 10.1186/s12870-017-1111-6

**Published:** 2017-10-16

**Authors:** Mei-Ya Liu, Asdrubal Burgos, Lifeng Ma, Qunfeng Zhang, Dandan Tang, Jianyun Ruan

**Affiliations:** 1 Key Laboratory of Tea Plant Biology and Resources Utilization (Ministry of Agriculture), Tea Research Institute, Chinese Academy of Agricultural Sciences, Hangzhou, 310008 China; 2 Max Planck Institute of Molecular Plant Physiology, 14476 Potsdam-Golm, Germany; 3 Graduate School, Chinese Academy of Agricultural Sciences, Beijing, 100081 China

**Keywords:** Lipidomics, TAG, MGDG, DGDG, Flavor/aroma origin compounds, Tea plant

## Abstract

**Background:**

Nitrogen (N) plays an important role in the formation of tea quality-related compounds, like amino acids and flavor/aroma origin compounds. Lipids, which have been reported to be affected by N deficiency, are precursors to the generation of flavor/aroma origin compounds in tea plant. However, there is no literature about the lipid profiles of tea plant affected by N fertilization. Hence, we hypothesize that the biosynthesis of flavor-related compounds in tea was affected by N through its regulation of lipid metabolism.

**Results:**

In this study, mature leaves and new shoots of tea plant grown under three N levels at the rates of 0, 285 and 474 kg/ha were applied for ultra-performance liquid chromatography-mass spectrometry (UPLC/MS) based lipidomic analysis. Totally, 178 lipid species were identified. The results showed that the composition of lipid compounds in mature leaves and new shoots varied dramatically, which was also affected by N levels. The higher content of the storage lipid TAG and higher carbon (C)/N ratio in mature leaves than that of new shoots in tea plants grown under low N level (0 kg/ha) suggested that tea plants could remobilize the C stored in TAG to maintain their C/N balance and help to improve the quality of tea. N fertilization resulted in a higher content of the compounds 36:6 MGDG and 36:6 DGDG. Since these compounds contain linolenic acid (18:3), a precursor to the formation of aroma origin compounds, we suggested their increase could contribute to the quality of tea.

**Conclusions:**

Taken together, the present work indicated that appropriate application of N fertilizer could balance the lipid metabolism and the formation of flavor/aroma origin compounds, which help to improve the quality of tea. Moreover, excess N fertilization might deteriorate the aroma quality of made tea due to increases of precursors leading to grassy odor.

**Electronic supplementary material:**

The online version of this article (10.1186/s12870-017-1111-6) contains supplementary material, which is available to authorized users.

## Background

Nitrogen (N) is one of the primary macronutrients essential to plants and is an important constituent of proteins, nucleic acids, phospholipids, chlorophyll, as well as the hormones that determine plant growth and development [1, 2]. Tea plant, a perennial evergreen shrub that is classified within the genus *Camellia* in the family Theaceae, contains abundant primary and secondary metabolites including free amino acids, flavonoids, and caffeine. These metabolite compounds are related to tea quality; their relative proportions determine the characteristics of made tea [3]. Numerous previous studies have shown that the biosynthesis of these primary and secondary metabolites is related to nutritional conditions [4–6]; tea plants grown at different N levels have distinct growth performance as well as different compositions of compounds related to quality. Good quality tea is produced, for example, when plants are grown at N levels between 200 kg/ha and 350 kg/ha [4, 7, 8].

Lipids are also major subcellular components that have been shown to play a number of key roles in plant growth, development, and responses to environmental cues [9, 10]. Glycerolipids are the most abundant lipid type in plants, comprising phospholipids, glycolipids and neutral lipids [10]. The first of these subgroups includes the compounds phosphatidylcholine (PC), phosphatidylethanolamine (PE), phosphatidylserine (PS), phosphatidylglycerol (PG) and phosphatidylinositol (PI), while the glycolipid group includes monogalactosyldiaclyglycerol (MGDG), digalactosyl diacylglycerol (DGDG) and sulphoquinovosyl diacylglycerol (SQDG), and diacylglycerol (DAG) and triacylglycerol (TAG) are both neutral lipids. The composition of these compounds differs between plant cellular membranes, tissues and species, and is also strongly influenced by nutrients [11, 12]. In the model plant system *Arabidopsis*, for example, lipid biosynthesis has been shown to be significantly affected by N nutrition [13, 14], while lipid remodeling is regulated by phosphorus starvation [15]. In addition, N limitations have also been shown to have a significant overall effect on the lipid composition of algae [16].

Lipids in the fresh leaves of tea plants are thought to be responsible for the generation of flavor and aroma compounds [17, 18]. (Z)-3-hexenol, its esters and (E)-2-hexenal, which principally contribute to the fresh and greenish odor of green tea, are oxidation products of free fatty acids formed during the tea manufacturing process. Hexenals and hexenols can also act as precursors to the formation of other compounds that generate aroma when tea is made; thus, as the de novo precursors for the biosynthesis of hexenals/haxenols, changes of lipids in tea plant will further affect the formation of aroma-generating compounds and thus the final quality of made tea. Even though several works point out lipid degradation during the process of tea manufacturing [19–21], lipid profiling had not been carried out in tea plant and nor the effects of N on tea lipids.

In this study, ultra-performance liquid chromatography-mass spectrometry (UPLC/MS) based lipidomics analysis [22] was applied for lipid profiling in the leaves of tea plants grown under three different N levels. This method has been successfully used to characterize the differential lipid remodeling that occurs during cold acclimation in natural accessions of *Arabidopsis thaliana* [23] and as well as phosphorus starvation [24]. Thus, the aims of this study were to: 1) Characterize the lipid composition of tea plant; 2) Describe the differences in lipid composition between mature leaves and new shoots, and; 3) Assess the changes of lipid species regulated by N fertilization, as some of which would further affect the quality of made tea.

## Methods

### Plant material and experiment

Samples of new shoots and mature leaves used in this study were collected in late March 2015 (the spring tea harvest season) from a long-term N addition field experiment owned by Tea Research Institute, Chinese Academy of Agricultural Sciences since 2006. The experimental tea plantation was established from rooted-cuttings of cultivar Longjing 43, a nationally released and commercially available clone optimum for premium green Longjing tea produce. The rates of N fertilizer were set as 0 (N1), 285 (N3), 474 (N4) kg/ha per year in four split applications i.e. early Feb (30% of total), May (20%), June (20%) and October (30%). Yields of new shoots in the previous years (2008–2014) were significantly increased by N applications while the yield increment was insignificantly different between N3 and N4 (Ma et al., personal communication). New shoots (one bud and two leaves) and the first mature leaves near the new shoots of tea plants grown under the above three N levels were sampled, then quickly frozen in liquid nitrogen, and stored in a − 80 °C ultra-refrigerator for lipidomic analysis and determination of carbon (C) and N contents. Samples for lipidomic analysis were lyophilized before taken to Max Planck Institute of Molecular Plant Physiology (Germany) for lipid measurement.

### Lipid extraction

Lipids were extracted from three biological replicates of mature leaves and new shoots of tea plants using the protocol described by Giavalisco et al. [22]. In brief, aliquots of 50 mg for each sample were prepared in the 2 ml Eppendorf tubes under constant freezing. Each sample was then suspended in 1 ml MTBE buffer. Extraction was performed on an orbital shaker for 10 min at 4 °C after incubating the samples in an ultrasonication bath for 10 min. Following this step, 0.5 ml of a mixture of water and methanol (3:1) were added and mixed well, then the tubes were centrifuge at 14000 rpm for 5 min in a table-top centrifuge at room temperature. The top organic phase containing lipid compounds was desiccated overnight, and keep frozen before measurement.

### Lipid fraction measurement using UPLC/MS

The lipids extracted from the previous step were separated on a UPLC system using a C_8_ reversed-phase column (100 mm * 2.1 mm * 1.7 μm particles; Waters) and the column temperature is 60 °C as established by Giavalisco et al. [22]. The mobile phases were water (UPLC MS grade; BioSolve) with 1% 1 M NH_4_Ac, 0.1% acetic acid (Buffer A,) and acetonitrile: isopropanol (7: 3, UPLC grade; BioSolve) containing 1% 1 M NH_4_Ac, 0.1% acetic acid (Buffer B). A 2 μl sample (the obtained organic fraction was re-suspended in 500 μl of acetonitrile: isopropanol 7: 3) was then loaded for each injection using a gradient of 1 min at 45% A, a 3 min linear gradient from 45% A to 35% A, an 8 min linear gradient from 25% A to 11% A, and a 3 min linear gradient from 11% A to 1% A. The injection flow rate was 400 μl min^−1^. The column was then washed for 3 min with 1% A, the buffer was set back to 45% A, and the column was re-equilibrated for 4 min. Thus, the total running time was 22 min.

An Exactive mass spectrometer (Thermo-Fisher) was applied to acquire the mass spectra. The spectra were recorded alternately between full-scan and all-ion fragmentation-scan modes, encompassing a mass range between 100 m/z and 1500 m/z. Resolution for all scans was set at 10000 and 10 scans were performed per second which restricted the loading time to 100 ms. The capillary voltage was set to 3 kV with a sheath gas flow value of 60 and an auxiliary gas flow of 35 (arbitrary units). The capillary temperature was set at 150 °C, the drying gas in the heated electrospray source was set at 350 °C, the skimmer voltage was set at 25 V, and the tube lens had a set avalue of 130 V. Spectra were recorded over the period between 1 and 17 min within UPLC gradients.

### Peak annotation, quantification and data analyses

GeneData software (Version 9.0, Refiner MS) was used to pre-process the chromatogram raw files; that is, baseline correction, chemical noise subtraction, chromatogram alignment, and peak detection. After preprocessing, a list of detected peaks and a matrix with their respective intensities for each sample were obtained (abs. File).

Peak annotation was carried out using an in-house developed R package grms (available upon request), based on the library compiled by Giavalisco et al. [22]. The software first performs a retention time (RT) correction of the output matrix based on previously identified markers with known RT. Then, the compounds are searched by comparing their specific m/z, expected adduct, and RTs within user-given ranges. A mass tolerance of 10 ppm was used to identify the lipid species. Peaks were confirmed by manually inspecting the chromatograms with the software Xcalibur (Thermo-Fisher). When isomers belonging to the same isobaric species were found they were denoted by adding a number within brackets after the isobaric species name.

Data normalization for lipid compounds detected by UPLC-MS was performed using R software. Lipids detected in positive and negative mode were combined before normalization. The coefficient of variation was fist calculated from raw chromatogram intensities for each compound. Then, the intensities of the compounds with 50% lower variation, excluding TAGs, were used as the normalization factor for all the compounds in the data set.

Statistical analyses were performed at the class level and the species level. For the class level, the content for every class was taken as the sum of species for the given class. Analysis of variance (ANOVA) and t-test (*P* < 0.05) were carried out at the two levels. Unsupervised principal component analysis (PCA) was run to obtain a general overview of the intrinsic variance of lipid profiles from different samples.

### Measurement of total N, total C and C/N ratio

To determine the total content of N, C and the C/N ratio in mature leaves and new shoots, an aliquot of 100 mg for each dried sample was used. The measurement was automatically finished in an elemental analyzer (Vario Max CN Analyzer, Elementar Analysensysteme GmbH, Germany).

## Results

### Lipids profiling in the leaves of tea plant

**Fig. 1:**
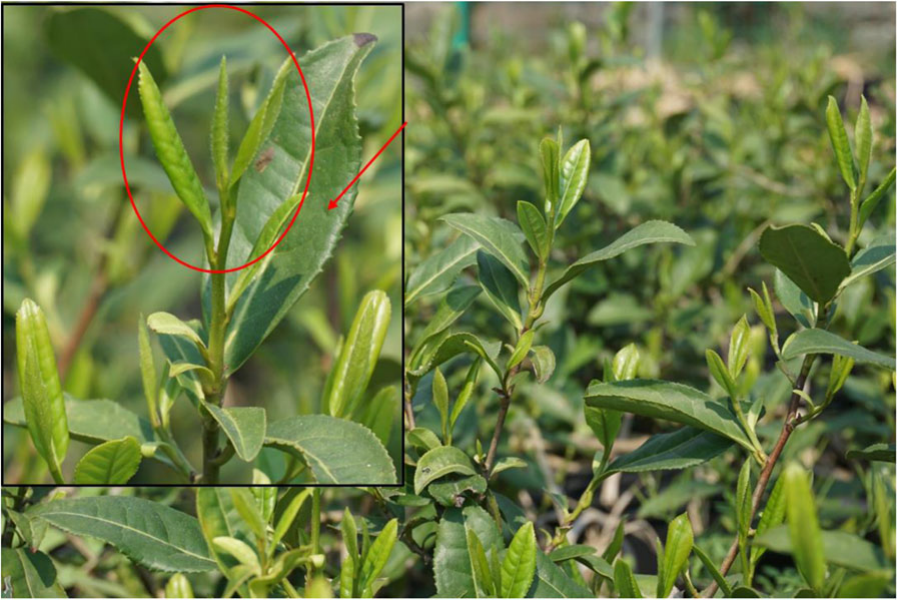
The ‘longjing43’ tea cultivar. The red circle indicated the new shoots of tea plant while the red arrow showed the mature leaves of tea plant

After measuring the lipid extracts from the mature leaves and new shoots of tea plants (Fig. 1), 178 lipid compounds were identified (Additional file 1: Table S1). All the main plant lipid classes were measured, with phospholipids being represented by PC (27 species), PE (26 species), PS (8 species), PI (2 species) and PG (6 species), and galactolipids being represented by MGDG (23 species), DGDG (21 species) and SQDG (13 species). The neutral lipid classes DAG and TAG have 8 and 44 species respectively.

**Fig. 2:**
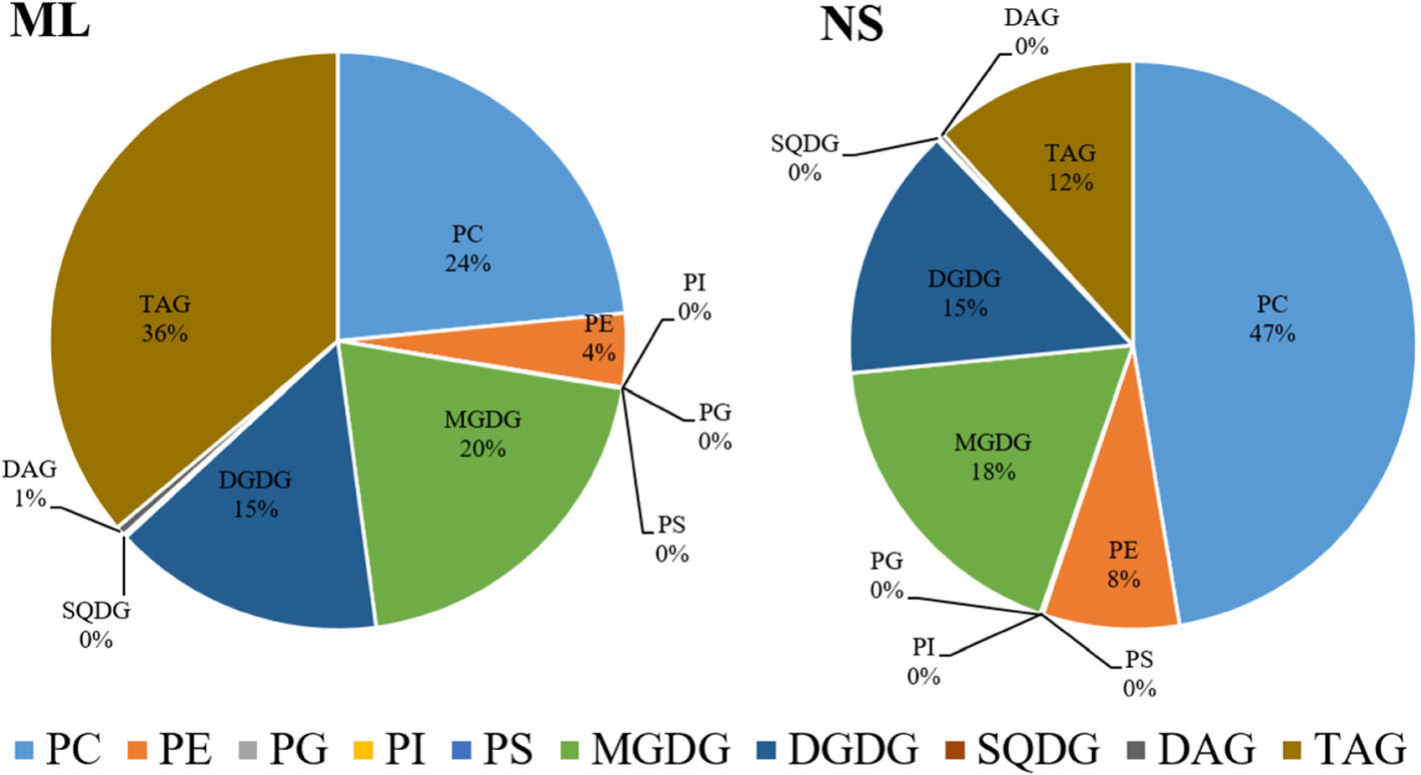
Composition of lipid classes in mature leaves (ML) and new shoots (NS) of tea plant. Values shown are the percentage contribution of (the sum of the mean intensities of all the compounds belonging to the same class) to (the sum of the mean intensities of all the classes). PC, phosphatidylcholine; PE, phosphatidylethanolamine; PS, phosphatidylserine; PG, phosphatidylglycerol; PI, phosphatidylinositol; MGDG, monogalactosyldiaclyglycerol; DGDG, digalactosyl diacylglycerol; SQDG, sulphoquinovosyl diacylglycerol; DAG, diacylglycerol; TAG, triacylglycerol

Lipid profiling from leaves, flower stalks, flowers, siliques, roots, and seeds of wild-type Arabidopsis confirmed that the content of lipid species differs among various plant organs [11]. In this study, two types of tea plant leaves (Fig. 1), mature leaves (ML) and new shoots (NS) were subjected to lipidomic analysis. As a result, the lipid composition varied between ML and NS groups (Fig. 2), the content of TAG accounted for 36% of all the identified lipids in ML group, while only 12% in the NS group. In contrast, PC compounds were highly abundant (47%) within the NS group, with that of 24% in ML group. Otherwise, the contents of PG, PI, PS and SQDG were rather low and the proportion of their contents was nearly zero among the whole contents of all the lipid compounds. The total content of DAG was higher in ML group than that of NS group, accounting for 1% and PC, PE, MGDG, SQDG, TAG were the main lipid classes both in the leaves of tea plant and Arabidopsis (Fig. 2).

It had been reported that changes in C/N ratio affected the accumulation of TAG [24] and the metabolism of lipids was also regulated by C:N availability [25]. Thus, the total contents of N, C, C/N were determined in this study (Table 1). Results showed that mature leaves had a higher level of C/N ratio than new shoots, while the total contents of N in new shoots were higher than that of mature leaves. Interestingly, the total C content was very similar in these two types of tea plant leaves (Table 1).

**Table 1:** Concentrations (mg/g) of total nitrogen (N), total carbon (C) and C/N ratio in mature leaves (ML) and new shoots (NS) of tea plant grown under three nitrogen levels at the rate of 0 (ML1/NS1), 285 (ML3/NS3), 474 (ML4/NS4) kg/ha (Mean ± SD, *n* = 4), respectively

	Total N	Total C	C/N ratio
ML1	29.2 ± 0.3	458.3 ± 0.4	15.7 ± 0.2
ML3	29.6 ± 0.7	454.6 ± 0.5	15.4 ± 0.4
ML4	33.3 ± 0.1	463.7 ± 0.6	13.9 ± 0.1
NS1	52.8 ± 2.3	469.7 ± 7.1	8.9 ± 0.5
NS3	53.7 ± 1.2	469.7 ± 9.9	8.8 ± 0.2
NS4	54.8 ± 2.7	465.5 ± 14.4	8.5 ± 0.3

### Principle component analysis of the lipid profiles

**Fig. 3:**
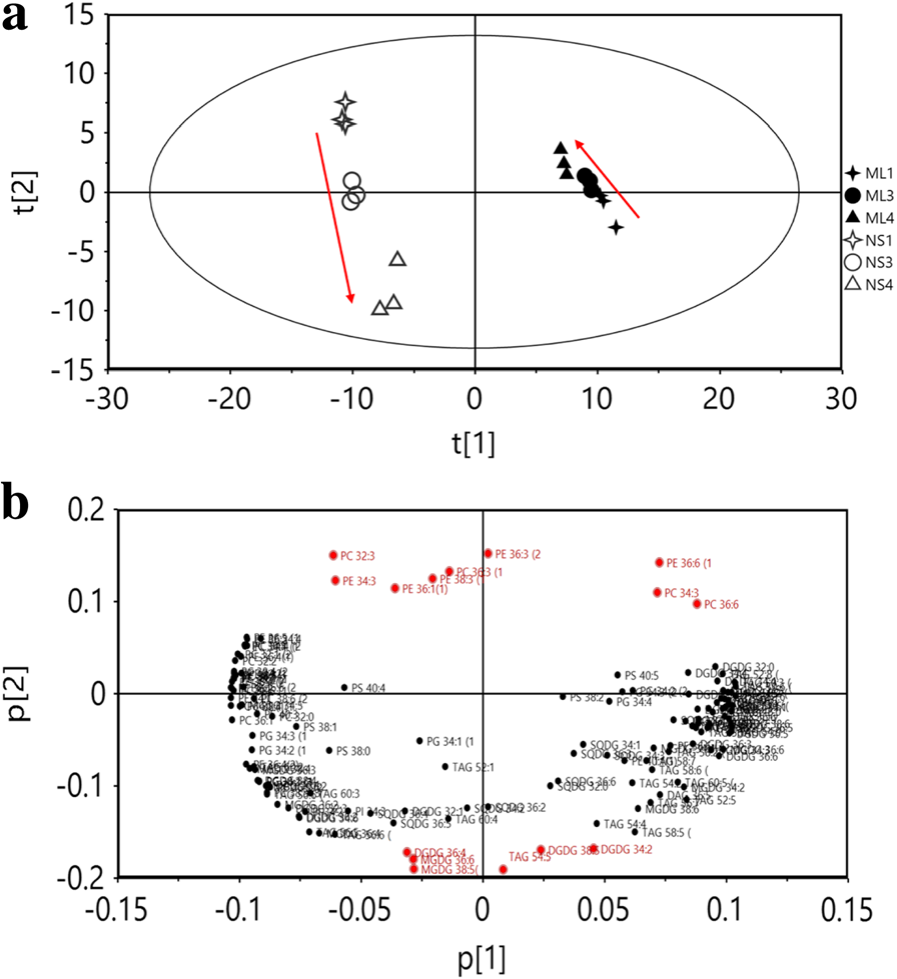
Principle component analysis of the lipid profiles. PCA score plot (**a**) and PCA loading plot (**b**) using lipid profiles from mature leaves (ML) and new shoots (NS) of tea plant grown under three nitrogen levels at the rate of 0 (ML1/NS1), 285 (ML3/NS3), 474 (ML4/NS4) kg/ha, respectively. The red arrow (**a**) indicated that the effects of nitrogen application on ML and NS were contrary. Lipids in red color (**b**) were the most responsive compounds under different nitrogen levels according to the loading values of principle component 2

Differences among the samples of mature leaves and new shoots harvested from tea plants grown under three N levels were determined via PCA. The PCA model explained more than 96.1% (R^2^) and predicted more than 88.4% (Q^2^) of the total variance. Principle component 1 (PC1) described the separation of lipids from ML and NS groups, while principle component 2 (PC2) explained the effects of N application (Fig. 3a). Lipids from the mature leaves and new shoots were apparently separated into two groups, while profiles from three different N levels were divided into three groups. Furthermore, the results also showed that new shoots were more significantly affected by N application than that of mature leaves and the effect of N application on these two tissues was contrary (Fig. 3a). According to the loading values of PC2, the changes of 32:3 PC, 34:4 PE, 36:1(1) PE, 38:3(1) PE, 36:3(1) PC, 36:3(2) PE in ML had positive correlations with the N application level, while 36:4 DGDG, 36:6 MGDG and 38:5 MGDG had negative correlations. In NS group, incremental application of N resulted in a negative effect on 36:1(1) PE, 34:3 PC and 36:6 PC, while a positive effect was shown in 38:5 DGDG and 34:2 DGDG lipid species (Fig. 3b, red colour compounds).

### The effect of N application on lipid composition in mature leaves and new shoots of tea plant

In this study, the application levels of N fertilizer in tea plantation were set as 0 (ML1/NS1), 285 (ML3/NS3), 474 (ML4/NS4) kg/ha, respectively. T-test (*P* < 0.05; Foldchange >2 and <0.5) was applied to distinguish lipids that were significantly affected by different N levels. In new shoots, a total of 36 lipid species were significantly changed at these two N levels (NS3 and NS4, both compared to NS1), among which most belonging to TAG class (26 species) (Table 2). The others were three SQDG species, two PE species, two PI species, two MGDG and only one DAG species. Comparing the NS3 level to NS1, 36:6(2) MGDG, 50:1 TAG, 52:1 TAG, 52:6 TAG, 52:7 TAG, 52:8(1) TAG and 54:9 TAG lipid species all underwent a 2 fold-change, while the contents of just one lipid species (34:4 PE) significantly decreased. At the N4 (higher N level) treatment condition, among the 36 significantly changed lipid species, the contents of the majority lipids increased more than 2 fold-change, except 34:4 PE and 36:6(1) PE with a decrease in their contents.

**Table 2:** Lipid species significantly affected by nitrogen in new shoots (NS) of tea plant. T-test (P < 0.05; Foldchange >2 and <0.5) was applied to separate the significantly changed lipids. The content of each lipid species under the three nitrogen levels was shown as (Mean intensity ± SD, SD = 3)

	NS1	NS3	NS4	Foldchange (NS3/NS1)	Foldchang (NS4/NS1)
34:4 PE	3.31 ± 0.49	0.78 ± 1.11	0.68 ± 0.96	0.24	0.21
36:6 (1) PE	128.7 ± 9.97	99.3 ± 10.75	64.14 ± 6.15	/	0.50
34:2 PI	1.16 ± 0.1	1.13 ± 0.81	2.85 ± 0.81	/	2.46
34:3 PI	1.74 ± 0.09	1.55 ± 1.1	3.76 ± 1.01	/	2.16
36:5 (1) MGDG	54.85 ± 3.09	77.54 ± 3.2	119.07 ± 0.18	/	2.17
36:6 (2) MGDG	7.82 ± 1.39	16.76 ± 2.74	30.25 ± 4.18	2.14	3.87
32:0 SQDG	0.31 ± 0.02	0.26 ± 0.18	0.78 ± 0.22	/	2.52
34:3 SQDG	7.93 ± 0.31	6.91 ± 4.88	20.69 ± 6.1	/	2.61
36:6 SQDG	1.67 ± 0.06	1.49 ± 1.05	4.33 ± 1.23	/	2.59
36:6 DAG	2.9 ± 0.33	4.9 ± 0.05	12.34 ± 0.72	/	4.26
50:1 TAG	3.38 ± 0.22	9.7 ± 0.57	6.92 ± 0.17	2.87	2.05
50:2 (1) TAG	31.54 ± 2.29	47.27 ± 4	75.38 ± 10.13	/	2.39
50:3 (1) TAG	26.87 ± 1.55	44.49 ± 3	74.97 ± 3.85	/	2.79
50:4 (1) TAG	1.83 ± 0.13	3.27 ± 0.42	5.8 ± 0.47	/	3.17
50:5 (1) TAG	1.02 ± 0.09	1.55 ± 0.07	2.96 ± 0.17	/	2.90
50:6(1) TAG	0.46 ± 0.04	0.63 ± 0.04	1.62 ± 0.21	/	3.52
52:1 TAG	1.32 ± 0.04	3.53 ± 0.19	2.12 ± 0.18	2.67	1.61
52:3 TAG	34.21 ± 1.43	58.45 ± 2.64	73.28 ± 4.25	/	2.14
52:5 TAG	200.08 ± 15.49	358.95 ± 8.85	644.61 ± 13.66	/	3.22
52:6 TAG	103.81 ± 10.36	199.37 ± 5.89	390.57 ± 18.38	1.92	3.76
52:7 TAG	1.27 ± 0.06	2.79 ± 0.22	5.26 ± 0.55	2.20	4.14
52:8 (1) TAG	0.06 ± 0.01	0.13 ± 0.02	0.33 ± 0.03	2.17	5.5
54:5 TAG	86.96 ± 7.62	142.32 ± 11.12	213.97 ± 4.9	/	2.46
54:9 TAG	59.84 ± 2.19	128.82 ± 7.67	272.83 ± 14.08	2.15	4.56
56:5 TAG	14.22 ± 0.64	21.73 ± 1.21	30.87 ± 2.97	/	2.17
56:6 (1) TAG	20.61 ± 1.37	33.82 ± 1.35	46.5 ± 4.54	/	2.26
56:6 (2) TAG	10.41 ± 1.44	16.78 ± 1.85	21.86 ± 2.93	/	2.10
56:7 TAG	13.23 ± 1.1	21.77 ± 0.98	33.35 ± 2.06	/	2.52
56:8 TAG	6.14 ± 0.77	9.58 ± 0.52	15.1 ± 1.28	/	2.46
56:9 TAG	1.71 ± 0.17	2.78 ± 0.1	5.12 ± 0.34	/	2.99
58:5 (2) TAG	2.72 ± 0.23	4.35 ± 0.19	6.85 ± 0.89	/	2.52
58:6 (1) TAG	3.95 ± 0.12	6.15 ± 0.69	8.84 ± 0.54	/	2.24
58:7 TAG	2.23 ± 0.21	3.33 ± 0.05	4.91 ± 0.45	/	2.20
60:5 (1) TAG	7.18 ± 0.51	11.29 ± 0.77	15.51 ± 0.83	/	2.16
60:6(1) TAG	4.53 ± 0.3	6.62 ± 0.42	9.29 ± 1.53	/	2.05
60:7(1) TAG	3.13 ± 0.13	4.61 ± 0.31	6.71 ± 0.35	/	2.14

In mature leaves, 21 lipid species were significantly affected by the application of N, which included two PC species, five PE species, and 14 TAG species (Table 3). At the ML4 (higher N level) treatment, only 32:0 PC, 32:1(2) PC, 36:1(1) PE, 36:6(2) PE, 38:1 PE accumulated, while their contents did not change at the ML3 N level (lower N level). The reduction of 38:3(2) PE occurred both under ML3 and ML4, whereas all the TAG lipid species were significantly decreased in ML4 and did not change at ML3.

**Table 3:** Significantly changed lipid species at different nitrogen levels in mature leaves (ML) of tea plant. T-test (P < 0.05; Foldchange >2 and <0.5) was applied to separate the significantly changed lipids. The content of each lipid species under ML1, ML3 and ML4 nitrogen levels was shown as (Mean intensity ± SD, SD = 3)

	ML1	ML3	ML4	Foldchang (ML3/ML1)	Foldchange (ML4/ML1)
32:0 PC	1.19 ± 0.15	1.61 ± 0.1	2.62 ± 0.26	1.35	2.20
32:1 (2) PC	1.61 ± 0.04	2.21 ± 0.38	3.39 ± 0.25	1.37	2.11
36:1 (1) PE	0.92 ± 0.12	1.82 ± 0.2	2.92 ± 0.12	1.98	3.17
36:6 (2) PE	0.18 ± 0.05	0.22 ± 0.04	0.75 ± 0.24	1.22	4.17
38:1 PE	0.15 ± 0.07	0.17 ± 0.02	0.39 ± 0.06	1.13	2.6
38:3 (2) PE	0.18 ± 0.04	0.03 ± 0.01	0.05 ± 0.03	0.17	0.28
38:4 (2) PE	0.2 ± 0.03	0.1 ± 0.03	0.14 ± 0.01	0.5	0.7
54:4 TAG	97.92 ± 4.52	61.47 ± 2.81	48.43 ± 1.67	0.63	0.49
56:4 TAG	28 ± 2.52	17.58 ± 0.6	13.23 ± 0.14	0.63	0.47
56:7 TAG	50.68 ± 1.56	31.16 ± 1.6	24.24 ± 0.48	0.61	0.48
56:8 TAG	64.85 ± 5.11	34.99 ± 2.91	23.21 ± 1.56	0.54	0.36
56:9 TAG	46.59 ± 0.79	25.57 ± 0.92	17.14 ± 0.07	0.54	0.37
58:4 TAG	65.37 ± 8.56	42.42 ± 1.53	32.49 ± 0.39	0.65	0.50
58:5 (1) TAG	20.68 ± 2.25	11.56 ± 0.51	7.4 ± 0.16	0.56	0.36
58:6 (1) TAG	18.57 ± 1.76	9.49 ± 0.32	6.77 ± 0.47	0.51	0.36
58:7 TAG	12.03 ± 0.36	6.16 ± 0.55	2.92 ± 0.07	0.51	0.24
60:2 TAG	3.27 ± 0.32	1.7 ± 0.08	1.37 ± 0.13	0.52	0.42
60:3 TAG	6.51 ± 0.65	3.9 ± 0.42	3.06 ± 0.16	0.60	0.47
60:4 TAG	13.9 ± 0.45	7.62 ± 0.54	6.19 ± 0.06	0.55	0.45
60:6 (1) TAG	40.18 ± 3.4	21.48 ± 1.97	15.84 ± 0.61	0.53	0.39
60:7 (1) TAG	59.83 ± 1.79	36.86 ± 1.51	25.95 ± 0.55	0.62	0.43

### Alterations of the main lipid classes in mature leaves and new shoots of tea plants under three N levels

**Fig. 4:**
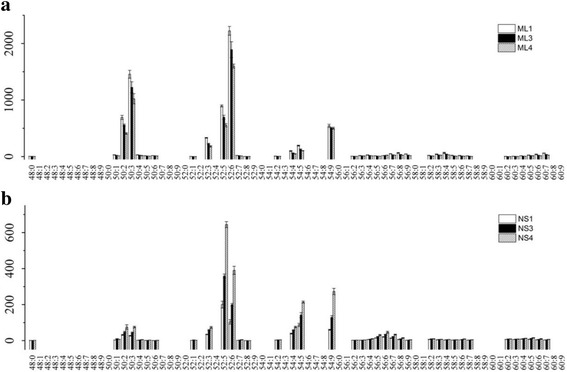
Alteration of TAG class in mature leaves (ML, **a**) and new shoots (NS, **b**) of tea plants grown under different nitrogen levels. Values shown were the mean intensities of each TAG species under three nitrogen levels at the rate of 0 (ML1 & NS1, white bar), 285 (ML3 & NS3, black bar) and 474 (ML4 & NS4, gray bar with oblique line) kg/ha, respectively. Error bars indicated the interval between the first and the third quartiles

**Fig. 5:**
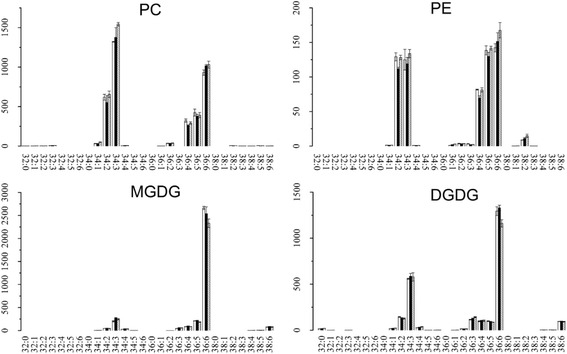
Changes of PC, PE, MGDG and DGDG classes in mature leaves (ML) of tea plants grown under different nitrogen levels. Values shown were the mean intensities of each species under three nitrogen levels at the rate of 0 (ML1, white bar), 285 (ML3, black bar) and 474 (ML4, gray bar with oblique line) kg/ha, respectively. Error bars indicated the interval between the first and the third quartiles

**Fig. 6:**
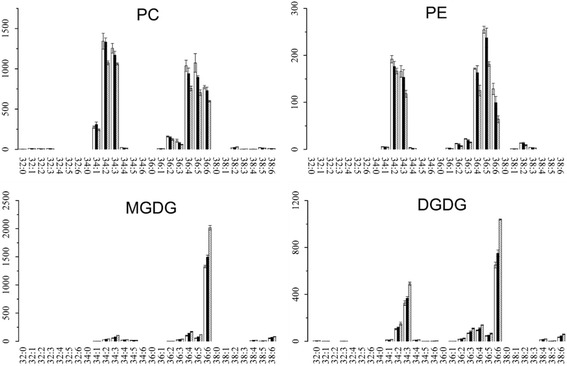
Changes of PC, PE, MGDG and DGDG classes in new shoots (NS) of tea plants grown under different nitrogen levels. Values shown were the mean intensities of each species under three nitrogen levels at the rate of 0 (NS1, white bar), 285 (NS3, black bar) and 474 (NS4, gray bar with oblique line) kg/ha, respectively. Error bars indicated the interval between the first and the third quartiles

As shown in Fig. 2, PC, PE, MGDG, SQDG, and TAG are the five major lipid classes in the leaves of tea plant. N deficiency usually leads to the accumulation of TAG, a response that is known to occur both in plants and green algae [12, 16, 26]. In mature leaves, the content of TAG decreased with the increment of N application level (Fig. 4a), which was consistent with the previous studies. Remarkably, TAG accumulated in the new shoots under high N level (Fig. 4b), in contrast to the previous studies in other organisms. There was little variation between high (ML4) and low N level (ML3) in mature leaves for the N containing lipid classes PC and PE (Fig. 5), while they both decreased at high N level in new shoots of tea plant (Fig. 6). N application did not affect the MGDG and DGDG lipid classes in mature leaves (Fig. 5 and Table 3) but induced the increment of their contents in new shoots, especially for 36:6 MGDG and 36:6 DGDG (Fig. 6 and Table 2). 36:6(2) MGDG increased by 2.14- and 3.87 fold-change at NS3 and NS4, respectively. Otherwise, the content of 36:6 SQDG was not significantly affected by NS3, but highly induced by 2.61 fold-change at NS4 N level.

## Discussion

In this study, the lipid profiles of tea plant *Camellia sinensis* L. were characterized and the effect of N fertilization on lipid composition was also uncovered. The composition of lipid compounds in mature leaves and new shoots varied dramatically (Fig. 2). Previous studies had confirmed that lipid composition differs between cellular membranes, plant tissues and plant species [11, 27]. Lipids are special compounds for tea plant, as they are precursors for tea aroma compounds [18]. So the lipid species in tea plant may have their own characteristics.

TAG is the most common lipid reserve in plants [25, 28, 29]. Studies in which N deficiency induced the accumulation of TAG had been well documented [12, 26, 30]. In the present work, the alteration of TAG in mature leaves of tea plant under zero, low- or high N levels (Fig. 4a) was consistent with these previous studies. In tea plant, mature leaves play the role as nutrient storage organ ‘sink’ and mobilise N to support the growth of new shoots when root uptake is limited due to low temperature in early spring [31]. Thus, the content of TAG in mature leaves was higher than that of new shoots in tea plants grown under low N level (Figs. 2 and 4), likely suggesting that tea plants could use the C stored in TAG to maintain their growth performance and also the shoots to sprout in early spring. With the increment of N application rate, TAG in mature leaves decreased (Fig. 4a) but increased in new shoots (Fig. 4b). MGDG, DGDG, SQDG and PG are the four unique lipids that composed of chloroplast lipids [32]. Their contents in new shoots increased with the increment of N fertilization level, while in mature leaves changes of their contents were not obvious under these three N fertilization level (Figs. 5 & 6). Indicating that the effect of N fertilization on chloroplast lipids in new shoots were more significant than mature leaves.

Moreover, it had been reported that the mobilization of storage lipids in Arabidopsis was regulated by C/N availability [24, 25]. In our study, the C/N ratio in mature leaves was higher than that of new shoots (Table 1) and the TAG class was highly accumulated in mature leaves of tea plant (Fig. 4a). Thus, in mature leaves, higher C/N ratio might induce the accumulation of TAG. The total content of N in mature leaves was lower than that of new shoots (Table 1) and the concentrations of N containing lipid classes PC and PE in mature leaves did not change both under high- or low N level (Fig. 5), while there is a decrease in new shoots (Fig. 6). It is likely that the changed C/N balance in new shoots was related to the process of C/N remobilization from mature leaves [33]. Interestingly, our work showed that while decreased N caused an accumulation of TAG in mature leaves, it caused its depletion in young shoots. Even though we did not have evidence explaining this founding in the present study, it could be speculated that TAG storage in mature leaves and new shoots had different purpose. More studies are needed to determine the physiological relevance of this finding.

The typical nomenclature for lipidomics goes as follows, number of carbons in both fatty acids and number of double bonds in both fatty acids. Linolenic acid (18:3) is the precursor for the formation of hexenals/hexenols [34–36]. In tea plant, the contents of hexenals/hexenols dramatically affect the flavor and aroma of teas [17, 18, 34]. Two highly abundant compounds varying significantly under the conditions tested could thus affect the quality of tea: 36:6 MGDG and 36:6 DGDG, each contains two 18:3 fatty acids. Compared to zero N application (NS1), their contents were dramatically increased under a higher N level (NS4) and with a slight increase at a moderate N level (NS3) (Fig. 6). Our previous results indicated that the optimum level of N fertilization for yield in the experimental plantation is N3 (285 kg/ha (Ma et al., personal communication)). The present results confirmed that higher level of N application is not good for tea plant, as higher N level increase the content of 36:6 MGDG and 36:6 DGDG (Fig. 6) in new shoots. In tea plant, usually the new shoots are harvested for the production of made tea [33]. So the alterations of 36:6 MGDG and 36:6 DGDG would possibly further increase the content of hexenals/hexenols and caused an undesirable grassy odour in made tea. Changes in fatty acid levels of new shoots of tea due to nitrogenous fertilizers explained the general flavour quality deterioration with increase in nitrogenous fertilizer rates [37, 38]. The lipidomics results in our present work suggested that changes in lipid composition which related to tea quality and aroma, were also affected by N application.

## Conclusions

In the present work, lipidomic analysis of the leaves in tea plant grown under three N application levels provided one of the first case studies, in which the lipid profiles of tea plant and the changes of lipids in the mature leaves and new shoots affected by N fertilization were uncovered. The lipid composition in mature leaves and new shoots was different, the later was more significantly affected by N. Otherwise, the mobilization of storage lipid TAG in mature leaves of tea plants grown under low N (0 kg/ha) level indicated that tea plants could reuse the C stored in TAG to maintain their growth performance and help to form a good quality of tea. Furthermore, precursors to the flavor/aroma origin compounds 36:6 MGDG and 36:6 DGDG were dramatically increased under a higher N level (474 kg/ha) and with a slight increase at a moderate N level (285 kg/ha), indicating that the rate of 285 kg/a nitrogen application is more suitable for tea plantation. All these confirmed that N fertilization contributes to the quality of tea through its regulation of lipid compositions.

## Additional file

Additional file 1: Table S1. All the identified lipid species in the leaves of tea plant (*Camellia sinensis* L.). (XLSX 17 kb)

## Abbreviations

C: Carbon
DAG: Diacylglycerol
DGDG: Digalactosyl diacylglycerol
MGDG: Monogalactosyldiaclyglycerol
ML: Mature leaves
N: Nitrogen
NS: New shoots
PC: Phosphatidylcholine
PC1: Principle component 1
PC2: Principle component 2
PCA: Principle component analysis
PE: Phosphatidylethanolamine
PG: Phosphatidylglycerol
PI: Phosphatidylinositol
PS: Phosphatidylserine
SQDG: Sulphoquinovosyl diacylglycerol
TAG: Triacylglycerol
